# Correction of Batch Effect in Gut Microbiota Profiling of ASD Cohorts from Different Geographical Origins

**DOI:** 10.3390/biomedicines12102350

**Published:** 2024-10-15

**Authors:** Matteo Scanu, Federica Del Chierico, Riccardo Marsiglia, Francesca Toto, Silvia Guerrera, Giovanni Valeri, Stefano Vicari, Lorenza Putignani

**Affiliations:** 1 Immunology, Rheumatology and Infectious Diseases Research Area, Unit of Microbiome, Bambino Gesù Children’s Hospital, IRCCS, 00165 Rome, Italy; matteo.scanu@opbg.net (M.S.); riccardo.marsiglia@opbg.net (R.M.); francesca.toto@opbg.net (F.T.); 2 Child and Adolescent Neuropsychiatry Unit, Bambino Gesù Children’s Hospital, IRCCS, 00165 Rome, Italy; silvia.guerrera@opbg.net (S.G.); giovanni.valeri@opbg.net (G.V.); stefano.vicari@opbg.net (S.V.); 3 Life Sciences and Public Health Department, Università Cattolica del Sacro Cuore, 00168 Rome, Italy; 4 Unit of Microbiology and Diagnostic Immunology, Unit of Microbiomics and Immunology, Rheumatology and Infectious Diseases Research Area, Unit of Microbiome, Bambino Gesù Children’s Hospital, IRCCS, 00165 Rome, Italy; lorenza.putignani@opbg.net

**Keywords:** machine learning, batch effect normalization, quantile regression, autism spectrum disorders (ASDs), gut microbiota, intestinal biomarkers

## Abstract

Background: To date, there have been numerous metataxonomic studies on gut microbiota (GM) profiling based on the analyses of data from public repositories. However, differences in study population and wet and dry pipelines have produced discordant results. Herein, we propose a biostatistical approach to remove these batch effects for the GM characterization in the case of autism spectrum disorders (ASDs). Methods: An original dataset of GM profiles from patients with ASD was ecologically characterized and compared with GM public digital profiles of age-matched neurotypical controls (NCs). Also, GM data from seven case–control studies on ASD were retrieved from the NCBI platform and exploited for analysis. Hence, on each dataset, conditional quantile regression (CQR) was performed to reduce the batch effects originating from both technical and geographical confounders affecting the GM-related data. This method was further applied to the whole dataset matrix, obtained by merging all datasets. The ASD GM markers were identified by the random forest (RF) model. Results: We observed a different GM profile in patients with ASD compared with NC subjects. Moreover, a significant reduction of technical- and geographical-dependent batch effects in all datasets was achieved. We identified *Bacteroides_H*, *Faecalibacterium*, *Gemmiger_A_73129*, *Blautia_A_141781*, *Bifidobacterium_388775*, and *Phocaeicola_A_858004* as robust GM bacterial biomarkers of ASD. Finally, our validation approach provided evidence of the validity of the QCR method, showing high values of accuracy, specificity, sensitivity, and AUC-ROC. Conclusions: Herein, we proposed an updated biostatistical approach to reduce the technical and geographical batch effects that may negatively affect the description of bacterial composition in microbiota studies.

## 1. Introduction

The identification of gut microbiota (GM) signatures associated with pathological and physiological conditions is crucial for understanding wellness and illness [1]. Indeed, the accurate identification of gut biomarker results is important to define host–microbiome interactions and to find new potential diagnostic and therapeutic targets [2]. Nowadays, numerous studies based on differential GM profiles of healthy subjects and patients have allowed the identification of key bacteria as biomarkers [1,3,4,5,6,7]. However, unravelling the intricate relationship between human host and microbial communities requires precise and accurate experimental designs and data analyses, as technical artefacts, such as experimental procedures, can confound biological interpretations of the results. A source of error can be identified in the batch effects that arise from differential processing of specimens and can lead to spurious findings and obscure true signals [8]. For example, the variability in sample processing, such as differences in sampling techniques or storage conditions, can influence microbial composition descriptions [8]. Moreover, different methods for DNA extraction, PCR amplification, and library construction [9], and also variations in sequencing depth, read quality, and other technical parameters due to platforms of sequencing, can impact the observed abundance of microbial taxa, leading to final biases and inconsistencies in data interpretation [10].

Furthermore, there is supporting evidence that geography plays a significant role in shaping gut microbiota composition [11,12]. Diet variability, driven by different levels of development, agricultural practices, and cultural traditions in different countries, is the primary factor for this phenomenon. The Western diet, prevalent in America and Europe, is typically low in fiber and high in fat and refined carbohydrates [11]. In contrast, the Eastern diet, which is common in China and neighboring countries, consists of rice or noodles, soup, and a variety of vegetable and meat dishes [11]. Other than these dietary differences, other lifestyle factors such as smoking habits, stress levels, and circadian rhythms also influence the intestinal microbiome [13]. Recognizing the geographic batch effect highlights the importance of considering territorial dietary and lifestyle patterns in gut microbiota research.

Hence, all together, technical artefacts and geographic variability may have the potential to overshadow true biological signals, leading to spurious associations and erroneous conclusions [14] and hindering the reproducibility of research findings, complicating efforts to validate study results across independent cohorts or replicate experiments in different laboratories [14]. Although attempts have been made to alleviate batch effects through standardized experimental designs [15], some confounding factors remain unavoidable in practice. Furthermore, in certain instances, the reasons for batch effects may be only partially or entirely unknown [16].

Moreover, another aspect to consider is the distribution of the metataxonomic data, which are typically highly zero-inflated, over-dispersed, and heterogeneous, with complex distributions.

In the literature, other statistical tools, such as ComBat, Limma, sva, and Harmony, have been proposed for batch effect corrections [17,18,19,20]. However, these methods are designed for single-cell RNA-Seq or microarray data and they are not suitable for adequately accounting for metataxonomic data, failing with sparse count data or zero-inflated datasets and overestimating batch effects in the presence of large or low-dimensional data.

Therefore, conditional quantile regression (CQR) represents the best method for metataxonomic data normalization, being able to enhance the robustness of analyses and uncover meaningful insights into the microbial dynamics underlying human health and disease.

For these purposes, we applied CQR for the reduction of batch effects from different datasets in the context of an important pediatric disease, i.e., autism spectrum disorder (ASD), to effectively identify, quantify, and mitigate batch effects, thereby improving the quality and reproducibility of GM results.

## 2. Materials and Methods

### 2.1. Patient Enrolment and Sample Collection

For the study, 123 patients with ASD aged 2–19 years (97 boys and 26 girls) were recruited at the Bambino Gesù Children’s Hospital (Rome, Italy), Italy, with a diagnosis of ASD based on the criteria of the *Diagnostic and Statistical Manual of Mental Disorders* (DSM-5) and confirmed by the Autism Diagnostic Observation Schedule (ADOS-2) and by the Autism Diagnostic Interview, Revised (ADI—R).

During clinical visits, a single fecal sample was collected from each patient. In total, 123 fecal samples were collected and stored at −80 °C, until following analysis at the Microbiome Biobank of the OPBG, node of the Biobanking and Biomolecular Resources Research Infrastructure of Italy (BBMRI) of the Human Microbiome Unit, until processing for GM metataxonomic analysis.

We divided our cohort into discovery (82 ASD plus 68 neurotypical control (NC)) and validation datasets (40 ASD and 41 NC) for the following analyses.

### 2.2. Gut Microbiota Profiling

The bacterial DNA was isolated from stool samples by a QIAmp fast DNA stool mini kit (Qiagen, Hilden, Germany) and quantified by a NanoDropTM 2000/2000c spectrophotometer (Thermo Scientific, Wilmington, MA, USA). The V3–V4 variable regions of the 16S rRNA gene were amplified by using the following primers: *16S_F 5′-(TCG TCG GCA GCG TCA GAT GTG TAT AAG AGA CAG CCT ACG GGN GGC WGC AG)-3′* and *16S_R 5′-(GTC TCG TGG GCT CGG AGA TGT GTA TAA GAG ACA GGA CTA CHV GGG TAT CTA ATC C)-3′*, as described in the MiSeq rRNA amplicon sequencing protocol (Illumina, San Diego, CA, USA). The PCR amplifications were set up as denaturation at 95 °C for 30 s, annealing at 55 °C for 30 s, and extension at 72 °C for 30 s, and a final extension step at 72 °C for 5 min, for 32 cycles, using a Fast Start Hifi Taq kit (Roche Diagnostics, Mannheim, Germany). DNA amplicons were purified by KAPA pure beads (Roche Diagnostics, Mannheim, Germany) and barcoded by unique index combinations by Nextera primers (Illumina, San Diego, CA, USA). The library amplicons were purified, quantified by a Quant—iT™ PicoGreen^®^ dsDNA assay kit (Thermo Fisher Scientific, Waltham, MA, USA), pooled, and diluted to a final concentration of 7 nM before sequencing on an Illumina MiSeqTM platform (Illumina, San Diego, CA, USA).

### 2.3. Bioinformatic Pre-Processing and Statistical Analyses for ASD-NC Comparison

For the comparison of ASD versus NC GM profiles, we selected 82 ASD and 68 age-matched NC profiles downloaded from the BioProject PRJNA280490 (Figure 1). Therefore, 150 fastq files were pre-processed in QIIME2 v.2023.2, and with the DADA2 plugin. Amplicon sequence variants (ASVs) were produced and successively assigned taxonomically by the Greengenes2 algorithm using the Greengenes nucleotide sequence database v2022.10.

Age and gender were evaluated as confounding factors by means of the microbiome multivariable association with linear model 2 (MaAsLin2) algorithm [21].

Ecological analyses were conducted on absolute abundances normalized through the random subsampling observations (rarefaction method) based on the minimum sample depth. In the alpha diversity analysis, the microbial diversity of each sample was quantified with Shannon–Wiener, Simpson, and Chao1 indexes, and the Mann–Whitney test was applied for group comparisons. In the beta-diversity analysis, the PERMANOVA test was applied to the distance matrix calculated with the Bray–Curtis dissimilarity algorithm.

The linear discriminant analysis (LDA) effect size (LEfSe) method [22] was performed on ASVs, occurring in at least 25% of the samples, with a relative abundance > 0.001 to identify bacterial genera differentially expressed among groups (*p*-adjusted ≤ 0.05 after FDR correction, LDA score ≥ 3.0).

### 2.4. Dataset Collection

Seven cross-sectional studies characterizing the GM composition of patients with ASD compared with neurotypical controls (NCs) and one dataset composed of 146 pediatric NCs (PRJNA280490), were selected from the PubMed NCBI database, selecting projects exclusively based on the V3–V4 sequencing of the 16S rRNA hypervariable regions on the MiSeq Illumina platform. Raw sequence data belonging to PRJEB45948 [23], PRJEB29421 [24], PRJNA578223 [25], PRJNA624252 [26], PRJNA769228 [27], PRJNA813424 [28], and PRJNA754695 [29] were download from the European Nucleotide Archive (ENA, https://www.ebi.ac.uk/ena/browser/home, accessed on 1 October 2023) and the Sequence Read Archive database (SRA, https://www.ncbi.nlm.nih.gov/sra, accessed on 1 October 2023).

The fastq files generated in this study were submitted to NCBI with code PRJNA1136218. The OPBG-produced fastq files of PRJNA754695, PRJNA280490, and PRJNA1136218 were merged to obtain a larger matrix. (Figure 1).

### 2.5. Bioinformatic Pre-Processing of the Overall Fastq Files

The fastq files were imported separately into QIIME2 v2023.2 [30] from each dataset, resulting in a total of 1356 fastq files (678 paired-end fastq files) (Figure 1). The DADA2 plugin [31] was used to denoise and filter out the chimeras from the paired-end reads. The resulting reads were then joined into ASVs. All forward and reverse reads were trimmed at a Phred Score >20 and merged with an overlap of 12 nucleotides, to avoid any bias introduced by differences in quality check (QC) and denoising/merging process parameters. The sequences were taxonomically assigned by querying the Greengenes nucleotide sequence database v2022.10 [32], using the greengenes2 plugin. The rooted phylogenetic tree was constructed using the q2—phylogeny align—to—tree—mafft—fasttree plugin.

The bacterial count matrices (ASV tables), taxonomy data frames, and phylogenetic trees were exported from QIIME2 and analyzed using R v4.3.2 for statistical purposes.

### 2.6. Merging Databases and Batch-Effect Correction

To reduce the technical batch effect due to different laboratory origin, the seven count matrices were joined by geographic origin, obtaining a total of three different national ASV matrices: Chinese dataset (three ASV matrices), Italian dataset (three ASV matrices), and Korean dataset (one ASV matrix) (Figure 1). These count matrices were further combined to create a unique ASV matrix with 3996 ASVs (whole dataset [WD]) to reduce both technical and geographical batch effects.

Each dataset was normalized using the conditional quantile regression (CQR) method with the ConQur R package v2.0 [8] (Figure 1).

The regress out-of-batch effect was performed by two steps as follows:

- Regression step.

Firstly, the linear regression determines the likelihood of each taxon’s presence to robustly estimate the theoretical count distribution without batch effect.

The algorithm’s model assumes that the probability of observing a non-zero [8]:

Yi (Yi, π = P (Yi > 0|Xi)

where Xi = (ZiT, BiT) = concatenate p-dimension covariates, following a logistic regression model [8]:

logit {P(Yi > 0|Xi)} = ZiTζ + BiTϒ

Bi = batch variable; Zi = set of characteristics, which includes the key variables based on prior knowledge, such as clinical, demographic, and genetic features. ConQur requires the inclusion of key variables because they play similar roles in the batch effect removal procedure.

ζ and ϒ are the true logistic coefficients associated with the covariates and batch variables, respectively.

Secondly, the linear quintile regression performed for each sample Xi the quintile normalization on the original count distribution of taxon, obtaining an estimate batch-free distribution. The linear quantile regression normalized the non-zero Yi as follows [8]:

Qwi (τ|Xi,Yi > 0) = ZiTα (τ) + BiTβ (τ)

where Wi|Yi > 0 = Yi|Yi > 0 + U, U-Uniform(0,1), α(τ) and β(τ) are the true quantile coefficients at the τ-th quantile of Wi, which corresponds to a non-zero Yi.

- Matching step.

Each sample’s count, belonging to theorical distribution, was matched with the corresponding count of the batch-free distribution at the same quantile level and, then, it assumed the normalized value derived from the quantile distribution.

### 2.7. Performance Evaluation of the Batch Normalization Method

To assess the performance of the batch normalization method, we firstly calculated the dissimilarity matrix with the Bray–Curtis algorithm on the two ASV tables, normalized and not normalized. Then, we applied the PERMANOVA test on the normalized (R^2^_batch-free)_ and not normalized (R^2^_batch_) dissimilarity matrices, grouping samples by each BioProject (R^2^_auth_) and by ASD/NC stratification (R^2^_phen_). Finally, to quantify the effectiveness of the CQR method on the batch reduction, we calculated the delta variability (∆R^2^) by the difference between R^2^_batch_ and R^2^_batch-free_. Positive ∆R^2^ values indicated a reduction in dissimilarity, while negative values indicated an increase in dissimilarity.

To estimate the variability of the batch effect, the PERMANOVA_R2 test (ConQur R package) [8] was performed on the dissimilarity matrices obtained by applying the Aitchison distance algorithm to the ASV tables. The PERMANOVA_R2 function quantified the differences in ASV relative abundances, deriving both from the intrinsic characteristics of samples (e.g., ASD/NC stratification) (i.e., KeyVariable) and from the BioProject batch (i.e., BatchVariable), before (KeyVariable_batch_ and BatchVariable_batch_) and after (KeyVariable_batch-free_ and BatchVariable_batch-free_) the application of the batch normalization on all count matrices. The ∆KeyVariable (KeyVariable_batch_/KeyVariable_batch-free_) and ∆BatchVariable (BatchVariable_batch_/BatchVariable_batch-free)_ were compared to quantify the effectiveness of the CQR method on the batch reduction.

### 2.8. The Application of Machine Learning Algorithm

We further assessed the above described approaches for the reduction in batch effect with a machine learning (ML) algorithm. The random forest (RF) classification model was used to identify the most important bacteria able to classify samples in the ASD and NC groups. Firstly, the bacterial dataset was randomly divided into training and test sets (80% and 20%, respectively), using the combination of ceiling and random_ordered R functions. Secondly, the tuning step through iterative optimizations using the 10-fold cross-validation (10-fold CV) method and the trainControl function of Caret R package v6.0-94 was applied to find the optimal RF parameters. The optimization of additional parameters, such as ntree and mtry, was carried out using the expand.grid and train functions of the Caret R package. In the third step, the validation step of the RF model was obtained, generating a confusion matrix (confusionMatrix function) on the test set using the same R package. The confusion matrix was used to compute the performance of the RF model, measuring accuracy (ACC), sensitivity (or true positive rate (TPR)), and specificity (or true negative rate (TNR)) [33]:

ACC = TP + TN/TP + TN + FP + FN

TPR = TN/TN + FP

TNR = TP/TP + FN

TP—true positive; TN—true negative; FP—false positive; FN—false negative.

With the sensitivity and specificity metrics, the area under receiver optimization characteristic (AUROC) value was calculated. The highest AUROC value was translated in the better classification performance.

The classification of the importance features was achieved by the mean decreasing Gini coefficient [34].

### 2.9. Accuracy of CQR Method Evaluation

To test the accuracy of the CQR method, the top scoring ASVs, obtained by the RF method, were tested on the validation dataset (40 ASD and 41 NC profiles). We applied the same bioinformatic procedures described for the discovery dataset on raw sequences to obtain the ASV matrix. The selected top scoring ASVs were used as features to run a 10-fold cross-validation by the RF method. The accuracy, sensitivity, specificity, and AUC -ROC values were calculated.

### 2.10. CQR Validation Test

To validate the CQR method, we replaced the in-house Italian dataset (82 ASD plus 105 NC) with the validation dataset (40 ASD plus 41 NC). The obtained matrix was further combined with the Chinese, Korean, and the remaining Italian matrices to produce a unique ASV matrix (whole validation dataset (WVD)). The RF model was applied to the WVD normalized by CQR and to the non-normalized WVD.

The AUC-ROC, accuracy, sensitivity, and specificity values were obtained pre- and post-normalization to evaluate the performance of the method.

## 3. Results

### 3.1. Gut Microbiota Composition in ASD and NC Groups of In-House Dataset

Firstly, 82 patients with ASD were compared with 68 NCs. The average age of patients with ASD was 6.89 years (SD = ±3.46) and NC subjects was 7.41 years (SD = ±3.52). The gender ratio in the ASD group was 63 (76.83%) males to 19 (23.17%) females while, in the NC group, it was 39 (57.35%) males to 29 (42.65%) females.

The remaining 40 patients with ASD and 41 NCs were used for external validation of the methods and were not included in the GM compositional analysis.

From 82 patients with ASD and 68 NCs, a total of 15,782,713 reads and 2273 amplicon sequence variants were obtained.

Alpha diversity analysis showed a bacterial diversity between the ASD and NC groups, regardless of absence of statistical significance (*p*-value > 0.05) (Figure 2A–C), whilst for beta diversity, statistically significant dissimilarity was observed (PERMANOVA test, *p*-value < 0.05) (Figure 2D), suggesting different gut bacterial profiles between the ASD and NC groups.

Regarding the analysis of differential bacterial abundance, at the genus level, patients showed a high prevalence of *Phocaeicola_A_858004*, *Bacteroides_H*, *Faecalibacterium*, *Dialister*, *Roseburia*, *Parasutterella*, *Acetatifactor*, *Dysosmobacter*, *Parabacteroides_B_862066*, *Lawsonibacter*, *Haemophilus_D_735815*, *Enterocloster*, *Hungatella_A_128155*, *Anaerotruncus*, *Phocea*, *Butyricimonas*, and *Clostridium_Q_134516*, and a reduction in *Bifidobacterium_388775*, *Blautia_A_141781*, *Streptococcus*, *Collinsella*, *Copromorpha*, *Anaerobutyricum*, *SIO2C1*, *Nanosynbacter*, *Pauljensenia*, *Gemella*, *Lancefieldella*, *Gordonibacter*, *Bulleidia*, and *Corynebacterium* (Figure 2E).

We attempted to identify a specific GM signature associated with ASD by removing the potential effect of latent confounding variables (i.e., age and gender). Consequently, after using a general linear model, we identified that *Dorea A* and *Bifidobacterium* 38,775 were associated with both case–control and age variables, while *Mediterraneibacter* A 155,507 was specifically associated with age (Supplementary Figure S1). No ASVs were associated with the gender variable.

### 3.2. Main Characteristics of the Nine Selected Datasets

By combining the datasets from nine selected cross-sectional studies, we analyzed a total of 678 16S rRNA gene sequencing data from 387 patients with ASD and 291 NCs. The average age of the ASD and NC subjects was 6.3 and 6.6 years, respectively. The gender ratio in the ASD group was 285 males to 102 females, while, in the NC group, it was 145 males to 146 females (Table 1).

Joining datasets by geographical origin, the Italian dataset comprised 118 patients with ASD and 125 NCs, with mean ages of 6.8 and 7.9 years, respectively. The Chinese dataset consisted of 215 patients with ASD and 128 NCs, with mean ages of 5.6 and 5.4 years, respectively. The Korean dataset included 54 patients with ASD and 38 NCs, with mean ages of 8.5 and 6.5 years, respectively.

The sample sizes of these datasets ranged from 12 [28] to 198 [27] subjects. Moreover, in all studies, the V3–V4 hypervariable regions were used for bacterial library construction and sequenced on MiSeq Illumina sequencing platforms.

### 3.3. Reduction of the Technical Batch Effect on Datasets from Different Geographical Origins

To consider the geographical origin of ASD and NC sets, we evaluated the reduction in the technical batch effect on the Italian and Chinese datasets, separately. In the Italian dataset, the microbial dissimilarity was ∆R^2^_Italian-BioProject_ = 0 (R^2^_batch_ = 0.028 and R^2^_batch-free_ = 0.028) (Figure 3A,B). The bacterial dissimilarity between the ASD and NC groups was ∆R^2^_Italian-ASD/NC_ = −0.001 (R^2^_batch_ = 0.021 and R^2^_batch-free_ = 0.022) (Figure 3C,D). The same comparisons were carried out for the Chinese dataset, obtaining a ∆R^2^_Chinese-BioProject_ = 0.02 (R^2^_batch_ = 0.038 and R^2^_batch-free_ = 0.018) (Figure 3E,F) and ∆R^2^_Chinese-ASD/NC_ = −0.006 (R^2^_batch_ = 0.007 and R^2^_batch-free_ = 0.013) (Figure 3G,H), for ASD/NC stratification.

The PERMANOVA_R2 function for the Italian dataset resulted in ∆BatchVariable_Italian_ = 0.001 and ∆KeyVariable_Italian_ = −0.006 (pre-normalization: BatchVariable_batch_ = 0.013 and KeyVariable_batch_ = 0.01; post-normalization: BatchVariable_batch_ = 0.012 and KeyVariable_batch_ = 0.016). Concerning the Chinese dataset, the PERMANOVA_R2 function resulted in ∆BatchVariable_Chinese_ = 0.025 and ∆KeyVariable_Chinese_ = −0.011 (pre-normalization: BatchVariable_batch_ = 0.038 and KeyVariable_batch_ = 0.007; post-normalization: BatchVariable_batch_ = 0.013 and KeyVariable_batch_ = 0.018).

### 3.4. Reduction of the Technical and Geographical Batch Effect on the Whole Dataset

The same analysis was conducted on the whole dataset. The microbial dissimilarity was ∆R^2^_BioProject_ = 0.032 (R^2^_batch_ = 0.068 and R^2^_batch-free_ = 0.036) (Figure 4A,B). The bacterial dissimilarity between the ASD and NC groups was ∆R^2^_ASD/NC_ = −0.002 (R^2^_batch_ = 0.006 and R^2^_batch-free_ = 0.008) (Figure 4C,D).

The PERMANOVA_R2 function resulted in ∆BatchVariable = 0.006 and ∆KeyVariable = −0.008 (pre-normalization: BatchVariable = 0.053 and KeyVariable = 0.004; post-normalization: BatchVariable = 0.047 and KeyVariable = 0.012).

### 3.5. Bacterial Biomarker Identification by Geographical Origin by Random Forest Algorithm

The performance of the batch normalization method and the identification of bacterial biomarkers of ASD gut microbiota was obtained by applying the RF classifier to the Italian, Chinese, and Korean datasets, separately.

The model trained on the Chinese dataset provided a bacterial classification with accuracy, sensitivity, and specificity values of 0.897, 0.955, and 0.792 (Figure 5A). Moreover, the AUC-ROC value was 0.98, indicating a good performance of the model. This performance was comparable to the outcome achieved on the Italian and Korean datasets (Figure 5B,C). The model trained on the Italian dataset was characterized by an accuracy value of 0.813, a sensitivity value of 0.818, and a specificity value of 0.808, obtaining an AUC-ROC value of 0.83. Regarding the Korean dataset, we obtained an accuracy value of 1, a sensitivity value of 1, and a specificity value of 1, with an AUC-ROC value of 0.99, despite the smaller number of samples compared with the other datasets.

From the RF classifier analyses applied to each geographical dataset, we selected the 1st 25 most important features of each dataset. Comparing the feature lists, we showed that *Bacteroides_H*, *Blautia_A_141781*, *Faecalibacterium*, *Phocaeicola_A_858004*, *Bifidobacterium_388775*, *Alistipes_A_871400*, and *Gemmiger_A_73129* were shared in all datasets. This result indicated a strong fingerprinting of ASD on the GM profile that overcame the geographical origin of the dataset.

However, we identified 12 features only present in the Chinese dataset, and 9 features exclusively present in the Italian and Korean datasets (Figure 5D). The Chinese and Italian datasets shared four features, the Chinese and Korean datasets two features, and the Italian and Korean datasets shared five features.

### 3.6. Bacterial Biomarker Identification in ASD Regardless of Geographical Origin by Random Forest Algorithm

We combined all the bacterial count matrices (WD matrix) to train a new RF model to assess the impact of the increase in both dataset size and geographical origins on the performance of the model.

Therefore, the WD matrix was normalized using the CQR method, and 70% of the total samples were randomly selected for the training set. The new model classified the 1st 25 important genera with high values of accuracy (0.889), sensitivity (0.928), and specificity (0.827) (Figure 6A,B). Furthermore, the AUC-ROC value of 0.93 demonstrated the maintenance of the good predictive ability of the RF model despite an increase in datasets and the presence of geographical and technical batches. The good performance of CQR normalization was further confirmed by comparing the top 25 bacterial features with those resulting from other RF models obtained by geographical stratification of the datasets. Six out of twenty-five bacterial features were shared, including *Bacteroides_H*, *Faecalibacterium*, *Gemmiger_A_73129*, *Blautia_A_141781*, *Bifidobacterium_388775*, and *Phocaeicola_A_858004*.

### 3.7. Validation of the Six Top-Scoring ASVs and the CQR Method

For the feature validation, we tested the capability of the selected six bacterial taxa to classify the validation of the Italian dataset (40 ASDs and 41 NCs) in ASD and NC (Supplementary Figure S2A). The values of accuracy, sensitivity, specificity, and AUROC were calculated.

This model showed good accuracy (accuracy = 0.875), sensitivity (sensitivity = 0.9286), specificity (specificity = 0.8), and AUC-ROC (AUROC = 0.85) values (Supplementary Figure S2B), validating the previously obtained results.

For the CQR validation, the RF model, applied to the normalized WVD, performed a feature classification with an accuracy = 07281, sensitivity = 0.9118, specificity = 0.4565, and AUROC = 0.81 (Supplementary Figure S3A). The feature classification calculated for the not-normalized WVD was characterized by accuracy = 0.6842, sensitivity = 0.7595, specificity = 0.5143, and AUROC = 0.61 (Supplementary Figure S3B). The highest RF scores achieved after normalization validated the capability of the CQR method to harmonize the data.

## 4. Discussion

The false positive results in studies on the human microbiota may arise from a wide heterogeneity in the composition of the microbiota, and also from the use of different technical approaches, and may produce a low concordance amongst studies in this field. Then, the removal or reduction of the batch effects represents an interesting challenge for microbiome studies. Herein, we explored the effects of technical and geographical variations on the results from gut microbiota analyses and proposed a bioinformatic workflow to overcome these outcomes. The technical batch effect refers to differences in technical procedures, including data collection, sequencing technologies, sample preparation, and other experimental processes that differ from bench to bench. The geographical batch effect includes differences in gut microbiota composition originating from population-specific features, due to environmental factors, diet, local habits, and other socio-cultural characteristics. These batch effects may confound the true microbial signatures, resulting in an aberrant identification of the microbial profile associated with physiological or pathological conditions.

Therefore, we propose a strategy based on the application of appropriate open-source tools, which aims at reducing the batch effect amongst gut microbiota datasets of ASD and NC subjects, with different geographic (i.e., Italy, China, and Korea) and laboratory origins, obtaining a more robust microbial profiling method.

Before the integration and normalization processes of the datasets, we compared our original ASD data with a publicly available subset of NCs, observing two distinct ecological and taxonomic profiles and confirming a specific gut microbiota composition in patients with ASD. Our data showed high levels of *Phocaeicola_*A_858004, *Bacteroides_*H, *Faecalibacterium*, *Dialister*, *Roseburia*, *Parasutterella*, *Acetatifactor*, *Dysosmobacter*, *Parabacteroides_*B_862066, *Lawsonibacter*, *Haemophilus_*D_735815, *Enterocloster*, *Hungatella_*A_128155, *Anaerotruncus*, *Phocea*, *Butyricimonas*, and *Clostridium_*Q_134516, and a reduction in *Bifidobacterium_*388775, *Blautia_*A_141781, *Streptococcus*, *Collinsella*, *Copromorpha*, *Anaerobutyricum*, SIO2C1, *Nanosynbacter*, *Pauljensenia*, *Gemella*, *Lancefieldella*, *Gordonibacter*, *Bulleidia*, and *Corynebacterium.*

In particular, the increase in *Faecalibacterium*, *Bacteroides*, *Parabacteroides*, *Parasutterella*, *Phocaeicola, Haemophilus*, and *Clostridium* in patients with ASD was consistent with previously published findings [25,33,35].

Interestingly, *Faecalibacterium* is a late colonizer of gut microbiota in healthy subjects and is present at very low levels until childhood [36]. The high levels of *Faecalibacterium* in patients with ASD could indicate its gut premature colonization possibly at the expense of other beneficial bacteria such as *Bifidobacterium*, which was decreased in ASD [24]. Interestingly, in NC patients, the presence of *Bifidobacterium* was strictly influenced by age, with high abundances observed in the early years. This reinforces the crucial role of *Bifidobacterium* in colonizing the healthy infant gut shortly after birth, where it helps in the maturation of the immune system, facilitates the digestion of complex sugars in breast milk, and contributes to a eubiotic GM [37,38,39]. The early colonization by *Bifidobacterium* is associated with long-term benefits for metabolic health and protection against infections, highlighting its importance during the critical windows of early development [39].

Moreover, the increase in *Phocaeicola* (previously named *Bacteroides*) was already described in ASD [25]. *Phocaeicola vulgatus* was associated with the altered cortisol levels, which may be involved in the pathogenesis of ASD through the hypothalamic–pituitary–adrenal (HPA) axis pathway [40]. Moreover, this microorganism was positively correlated with the levels of D-glutamine and D-glutamate in the ASD gut metabolome [40]. We can conclude that high levels of *P. vulgatus* may contribute to the pathophysiology of ASD by affecting the neurotransmitter imbalance.

However, we showed the increase in Gram-negative bacteria, such as *Bacteroides, Parasutterella, Phocaeicola, Parabacteroides*, and *Prevotella*, which are lipopolysaccharide- (LPS-) producers. LPS has been found increased in the serum of patients with ASD and has been associated with impaired social behavioral scores [41]. LPS stimulates the secretion of proinflammatory cytokines from peripheral blood mononuclear cells and lymphoblasts of children with ASD [42], probably contributing to both peripheral and brain inflammation associated with the disease.

Interestingly, *Bifidobacterium* was decreased in ASD. This microorganism is a promoter of healthy status for its ability to produce neuromodulators and influence the gut–brain relationship through interaction with other commensal bacteria [43]. Recently, in a clinical trial, the administration of *Bifidobacterium* CCFM1025 in patients with major depression disorder (MDD) attenuated disease symptoms by regulating tryptophan metabolism and gut eubiosis [44], acting on the gut–brain axis.

Our bioinformatic pipeline, based on the application of CQR normalization on datasets assembled for geographical origin, resulted in being effective in reducing technical variations and improving the comparability of data across samples, strengthening the differences between ASD and NC groups. Furthermore, by the CQR method, we normalized each dataset according to the magnitude of the technical batches. Indeed, while the microbial dissimilarity amongst Chinese BioProjects was higher than amongst Italian BioProjects, indicating a high heterogeneity of the sequencing data from the selected Chinese BioProjects. After normalization, a higher reduction in microbial dissimilarity was observed in Chinese data than in Italian data. These results indicated that the initial technical batch effect was quantified properly by CQR, avoiding an over- or underestimation in the batch reduction. Moreover, these results were confirmed by the AUC-ROC prediction values, corroborating the improvement in prediction performance, and also addressing skewed distributions.

Interestingly, by this analysis, we identified *Bacteroides*, *Alistipes*, *Faecalibacterium*, *Gemmiger*, *Blautia*, and *Bifidobacterium* as biomarkers of ASD GM, independently from the geographical origin, showing that the microbiota features associated with ASD were not influenced by diet or socio-cultural origin.

Moreover, we characterized unique bacterial features in each dataset (Italian, Korean, and Chinese datasets). These observations underscored that the gut microbiota was specific to a distinctive population, not only inherently shaped by the host background but also by a disease-related phenotype. This factor must be carefully considered in any microbiota study.

The application of the CQR method on the WD also performed well, since we observed, with beta diversity analysis, a reduction in geographical batch effect, maintaining the differences between the ASD and NC groups. This evidence was consistent with the high performance of bacterial feature classification obtained by the AUC-ROC test. In fact, the inclusion of subjects with different geographical origins increased the degrees of batch effects. The RF model applied to the normalized data showed the accuracy values (i.e., AUC-ROC, specificity, and sensitivity values) comparable to those obtained separately by the normalization of the Italian and Chinese datasets. This evidence established the high robustness of the CQR method, which maintained its performance with a dataset characterized by a higher batch effect degree and sample size. Then, the reduction of the batch effect was critical to ensure accurate and reliable prediction, especially when integrating data from different sources. In addition, the WD analysis revealed that *Faecalibacterium*, *Bacteroides*, *Bifidobacterium*, *Blautia*, *Gemmiger*, and *Phocaeicola* were among the top 25 most important bacterial features. The consistency of the results obtained by different approaches (e.g., discrete geographical origin datasets vs. whole dataset) indicated an improvement in the robustness of analysis prediction by batch normalization.

Furthermore, these results agreed with other studies that reported *Faecalibacterium*, *Bacteroides*, and *Gemmiger* as biomarkers of gut microbiota of patients with ASD [45,46], while *Bifidobacterium* and *Blautia* were associated with NC children [47,48].

Interestingly, *Alistipes* was not confirmed by WD analysis. The explanation could be found in the dietary habits of patients [49]. Finally, the RF model assigned *Parasutterella*, *Prevotella*, and *Parabacteroides* as classifiers of the GM of patients with ASD, consistent with other studies [25,32,50].

Other than the concordance of our results with the literature, we tested the importance of these bacterial features that contributed to the case–control classification in another independent dataset, showing a good accuracy in prediction. Furthermore, we demonstrated that the application of the CQR method reduced the false positive and false negative rates.

Our results are promising, but there are some limitations. In order to obtain a dataset as homogeneous as possible, we used strict inclusion criteria to select datasets, such as pediatric age range, presence of sequences from both ASD and neurotypical subjects, selection of V3–V4 hypervariable region sequencing of the 16S rRNA, and choice of only MiSeq Illumina platform-based sequencing. Therefore, the resulting dataset was limited. Moreover, most of the raw sequences, deposited in public databases, did not provide metadata, as the most basic like gender and sex, and patient characteristics, like treatment, disease status, symptoms, diet, and lifestyle habits. For these reasons, it was not possible to perform an exhaustive analysis of confounding factors. Further studies on datasets enriched with more anamnestic variables will reduce statistical error rates, producing stronger correlations and validation of compositional and functional GM profiles for patients with ASD.

## 5. Conclusions

In conclusion, we reported an innovative, comprehensive, and robust bioinformatic strategy that reduces batch effects in different datasets. With our approach, we demonstrated an improvement in the AUC-ROC, accuracy, sensitivity, and specificity values obtained after normalization compared with those obtained before normalization. Thus, the use of the normalization method for the reduction in batch effects should be mandatory for ensuring the reliability and reproducibility of research findings in microbiota studies.

Moreover, for the first time, *Bacteroides_*H, *Faecalibacterium*, *Gemmiger_*A_73129, *Blautia_*A_141781, *Bifidobacterium_*388775, and *Phocaeicola_*A_858004 were identified as robust bacterial biomarkers of GM for ASD, regardless of geographical origin of the subjects, indicating a GM profile unaffected by dietary habits or socio-cultural differences. Unlike previous research, often confounded by environmental factors, our findings reveal biomarkers that remain consistent across diverse populations. This pivotal insight not only highlights the novelty of our work but also represents a major leap forward ASD diagnostics, offering more universal and reliable biological indicators, free of external variables’ influence.

Continued efforts to refine methods for batch effect reduction will be essential for advancing our understanding of microbial communities and their role in human health and disease. Ultimately, by adopting rigorous approaches to address batch effects, we can strengthen GM research and pave the way for more impactful standardization procedures in the field of microbiome science.

## Figures and Tables

**Figure 1:**
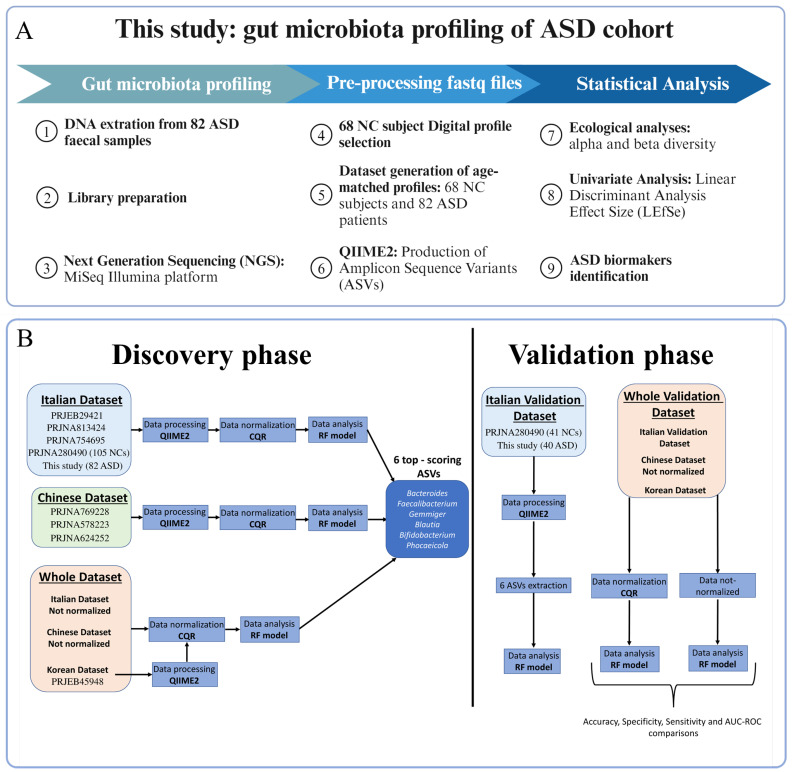
Graphical summary. (**A**) Scheme of the comparative workflow for ASD and NC groups. From 82 fecal samples, the bacterial DNA was extracted and the V3–V4 hypervariable region of 16S rRNA was amplified and sequenced on the MiSeq Illumina platform. Amplicon sequence variants (ASVs) were obtained from a total of 150 fastq files (82 ASD fastq files and 68 NC fastq file age, match-selected from PRJNA280490 BioProject) and were assigned taxonomically by the Greengenes database v2022.10. The ecological and univariate analyses were conducted for statistical comparisons. (**B**) Workflow of the batch effect correction. In the left panel (Discovery Phase), a selection of ASVs that classify an individual as either autistic or neurotypical control by applying conditional quantile regression (CQR) and random forest (RF) models to a 16S rRNA sequencing datasets. In the right panel (Validation Phase), validation of the selected set of ASVs and the CQR method using the Italian validation dataset and the whole validation dataset, respectively.

**Figure 2:**
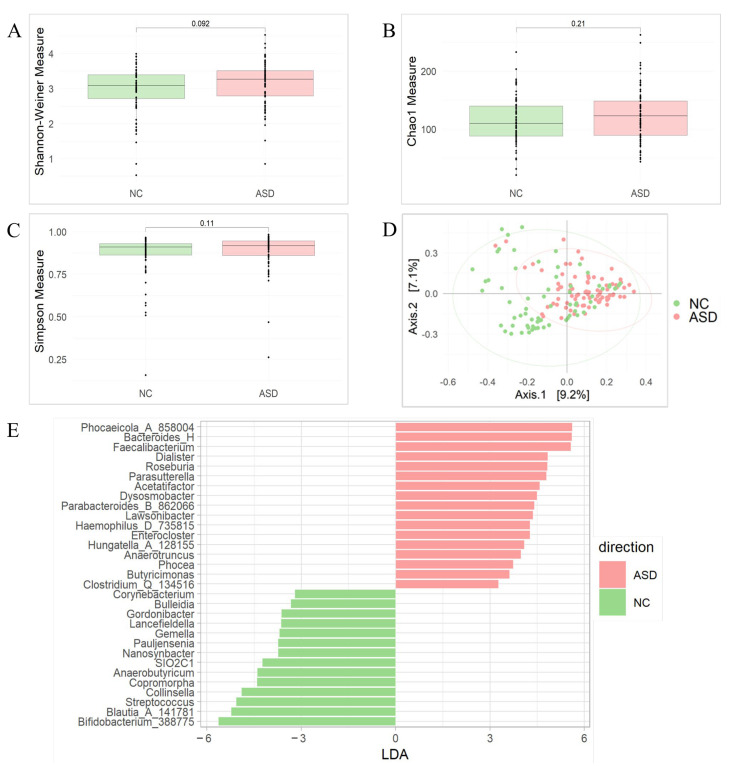
Comparison of gut microbiota composition between ASD and NC groups. Alpha diversity analysis was evaluated by Shannon–Wiener, Simpson, and Chao1 indexes, and the Mann–Whitney test was used to compare ASD (red) and NC (green) groups (*p*-value > 0.05) (**A**–**C**). Principal coordinate analysis (PCoA) shows the dissimilarity between ASD and NC groups calculated by the Bray–Curtis dissimilarity algorithm (PERMANOVA test, *p*-value < 0.05) (**D**). Univariate analysis performed with linear discriminant analysis effect size (LEfSe) shows genera differentially expressed and statistically significant between ASD and NC groups with an LDA value > 3 (*p*-adjusted < 0.05) (**E**).

**Figure 3:**
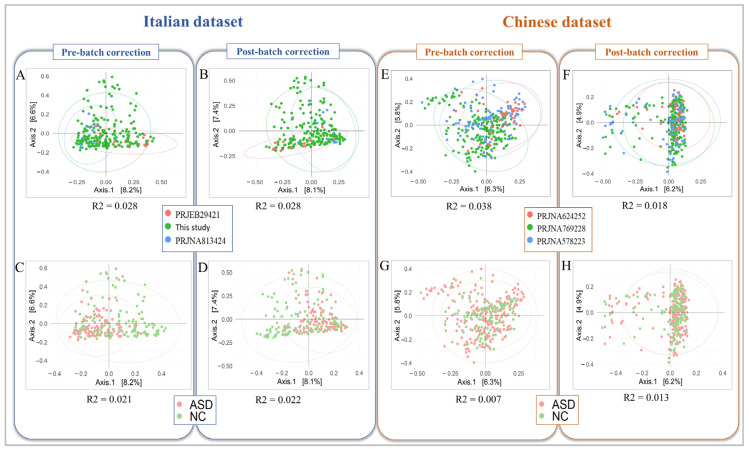
PCoA plot of the Bray–Curtis dissimilarity in Italian and Chinese datasets. Principal coordinate analysis (PCoA) was performed on dissimilarity matrices produced by the Bray–Curtis algorithm. In the left panel, the biplots show the PCoA applied to the Italian dataset pre- and post-technical batch correction for the comparison between BioProjects (**A**,**B**) and between ASD and NC groups (**C**,**D**). In the right panel, the biplots show the PCoA applied to the Chinese dataset pre- and post-technical batch correction for the comparison between BioProjects (**E**,**F**) and between ASD and NC groups (**G**,**H**). The R2 values, calculated by the PERMANOVA test, are statistically significant (*p*-value < 0.05).

**Figure 4:**
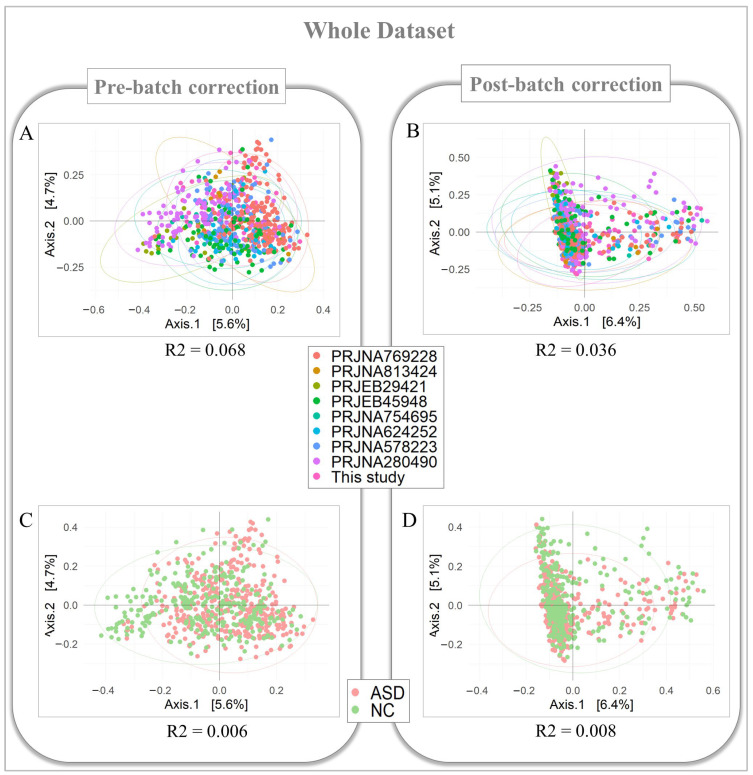
PCoA plot of the Bray–Curtis dissimilarity in whole dataset. Principal coordinate analysis (PCoA) was performed on dissimilarity matrices produced by the Bray–Curtis algorithm. The biplots show the PCoA performed on dissimilarity matrices pre- and post-technical and geographical batch normalized for the comparison between BioProjects (**A**,**B**) and between ASD and NC groups (**C**,**D**). The R^2^ values, calculated by the PERMANOVA test, are statistically significant (*p*-value < 0.05).

**Figure 5:**
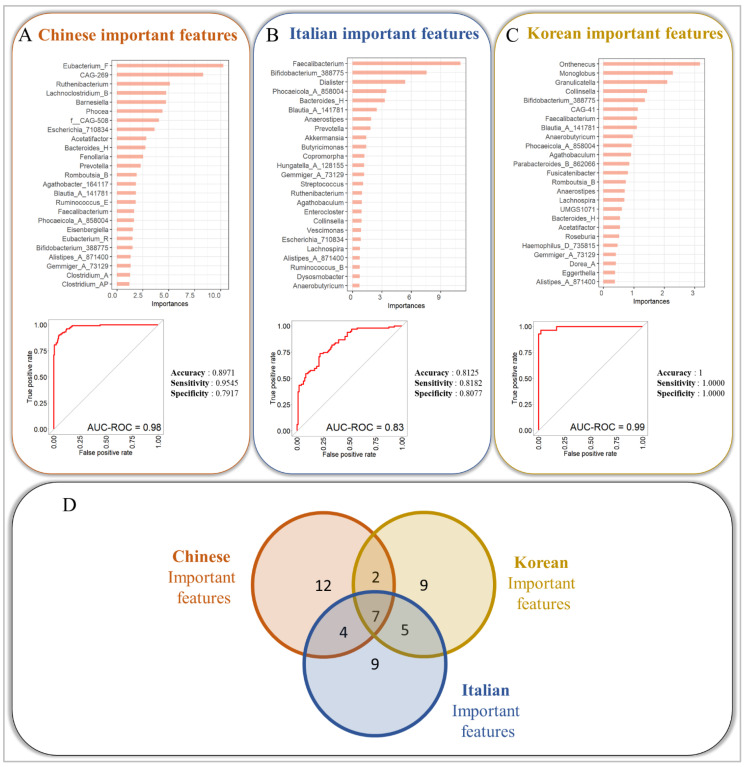
Random forest model applied to bacterial matrices merged by geographical origin. The importance of the 1st 25 genera in the predictive model applied to Chinese (**A**), Italian (**B**), and Korean (**C**) matrices were evaluated using the mean decreasing Gini coefficient. For each RF model, the accuracy, sensitivity, specificity, and AUC-ROC values are reported. The Venn diagram (**D**) shows the number of unique and shared most important features between datasets.

**Figure 6:**
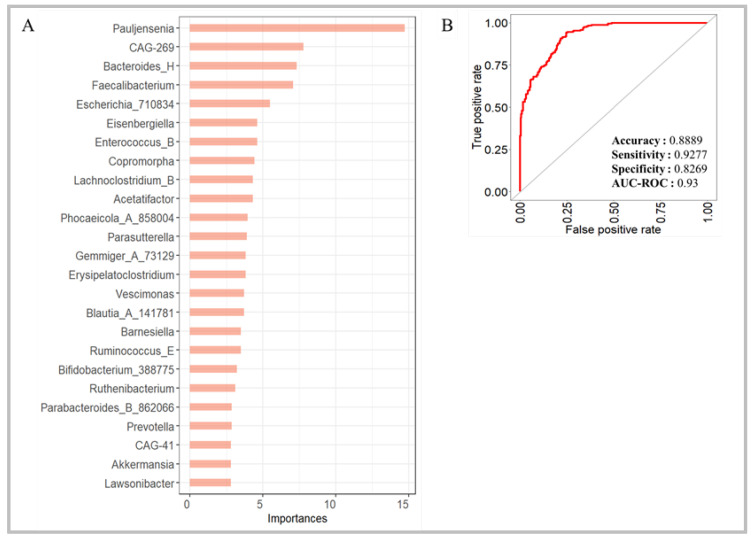
Random forest applied to the entire count matrix at the genus level. The importance of the 1st 25 genera in the predictive model was evaluated using the mean decreasing Gini coefficient (**A**). The accuracy, sensitivity, specificity, and AUC-ROC values of the RF model are reported (**B**).

**Table 1:** Characteristics of the selected datasets. Detailed information on the authors and year of publication, demographic characteristics, and diagnostic methods used for ASD.

BioProject	Country	ASD ^1^		NC ^2^		ASD Diagnostic Tools ^3^	Reference
		Number of Subjects	Age Average (Years)	Number of Subjects	Age Average (Years)		
PRJEB29421	Italy	11	3	14	3	DSM-5, ADOS-2, ADI-R, VABS, CARS	[24]
PRJEB45948	Korea	54	8.5	38	6.5	DSM-5, ADOS-2, ADI-R, SRS	[23]
PRJNA813424	Italy	6	14.5	6	15	DSM-5	[28]
PRJNA624252	China	29	4.4	20	4.3	ADOS-2, ADI-R	[26]
PRJNA769228	China	138	6.11	60	6.65	DSM-5, ADOS, CARS	[27]
PRJNA578223	China	48	5	48	4	DSM-4, ADI-R, CGI-S	[25]
PRJNA280490	Italy	n.a.	n.a.	105	8	n.a.	
PRJNA754695	Italy	19	7.16	n.a.	n.a.	DSM-5, ADOS-2, ADI-R	[29]
This Study	Italy	82	6.89	68	7.41	DSM-5, ADOS-2, ADI-R	

^1^ ASD—autism spectrum disorder; ^2^ NC—neurotypical control subjects; ^3^ DSM-5—*Diagnostic and Statistical Manual of Mental Disorders*, 5th Edition; ADOS—Autism Diagnostic Observation Schedule; ADI-R—Autism Diagnostic Interview, revised; VABS—Griffiths Mental Development Scales; CARS—Childhood Autism Rating Scale; SRS—Social Responsiveness Scale; DSM-4—*Diagnostic and Statistical Manual of Mental Disorders*, 4th Edition; CGI-S—Clinical Global Impression Severity of Illness scale, n.a.—not applicable. Sequencing platform is shared by all studies.

## Data Availability

The data presented in this study are openly available at https://www.ncbi.nlm.nih.gov/bioproject with accession number PRJNA1136218 accessed on 8 October 2024.

## Supplementary Materials

The following supporting information can be downloaded at: https://www.mdpi.com/article/10.3390/biomedicines12102350/s1, Supplementary Figure S1: Confounding factor analysis with MaAsLin2; Supplementary Figure S2: Validation of the six top scoring ASVs selected on the Italian Validation Dataset; Supplementary Figure S3: Validation of the CQR method.
