# Supplementary material for: Correction of Batch Effect in Gut Microbiota Profiling of ASD Cohorts from Different Geographical Origins
Source: Biomedicines. 2024 Oct 15;12(10):2350. doi: 10.3390/biomedicines12102350 (PMC11504477; doi:10.3390/biomedicines12102350)
Supplement: Supplementary file 1 [file biomedicines-12-02350-s001.zip › biomedicines-3197733-supplementary.pdf]

Supplementary Figures

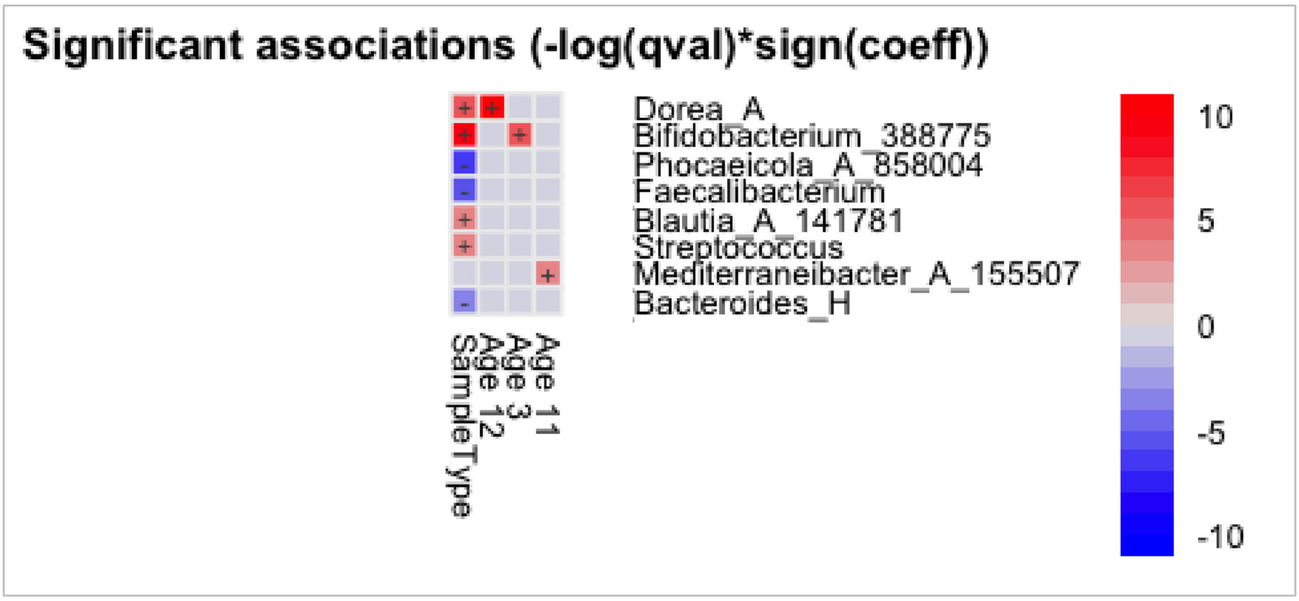

Supplementary Figure S1. Confounding factor analysis with MaAsLin2. The heatmap shows significant associations between ASVs and SampleType (case – control) and age variables. Red boxes indicate high association with NC groups and blue boxes indicate high association with ASD group. Grey boxes refer to the absence of statistical associations between ASVs and variables.

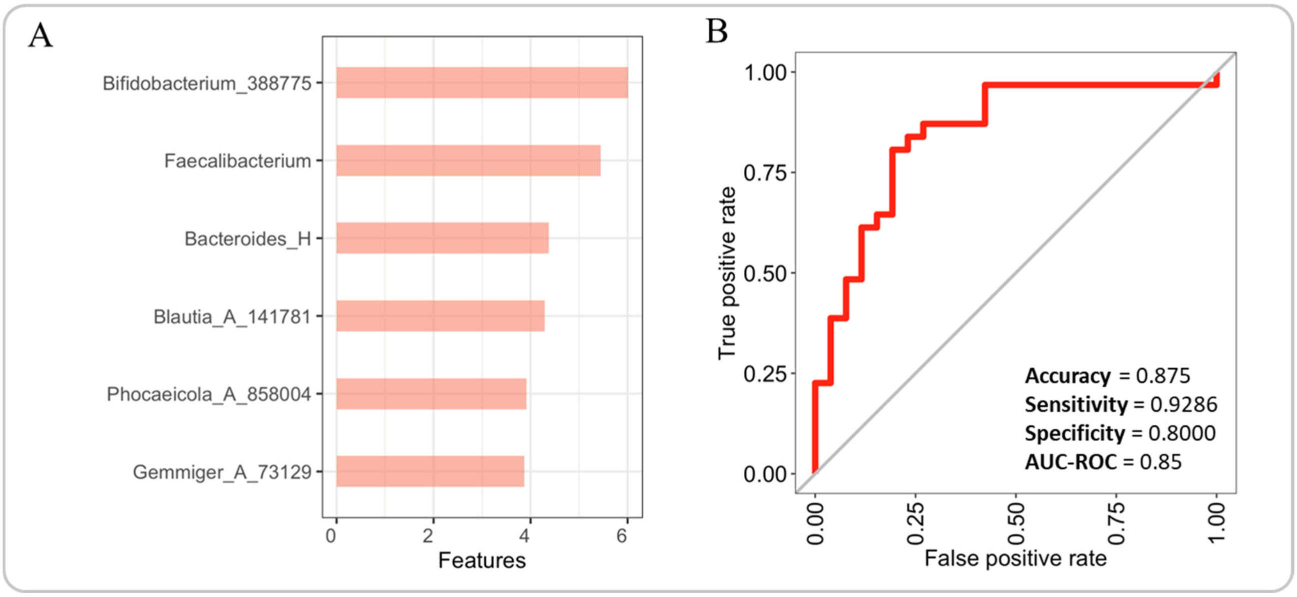

Supplementary Figure S2. Validation of the six-top scoring ASVs selected on the Italian Validation Dataset. The importance of the six top genera achieved with RF model applied on Italian Validation Dataset (A). The accuracy, sensitivity, specificity and AUC – ROC values are reported (B).

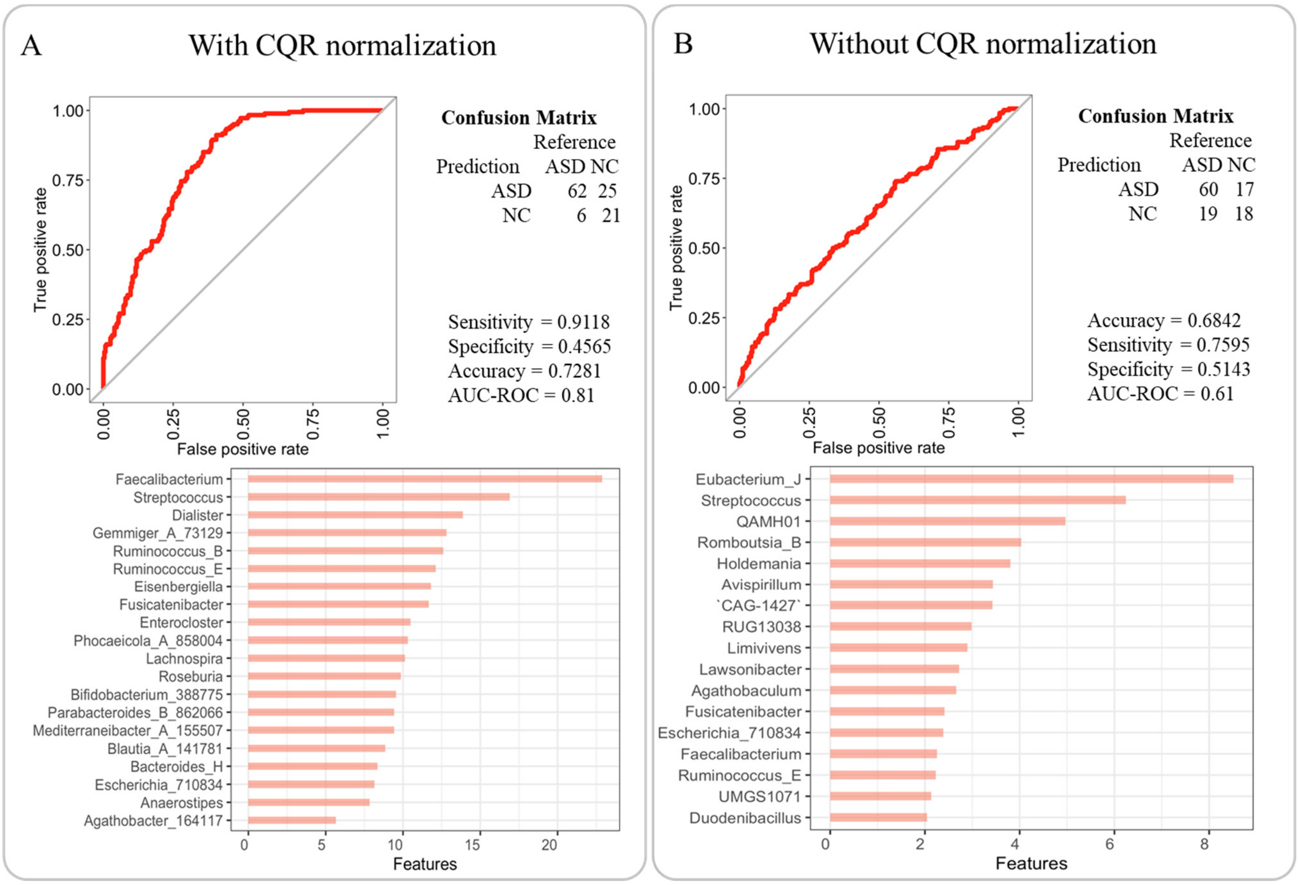

Supplementary Figure S3. Validation of the CQR method. The importance of the top scoring genus in the predictive model applied on the Whole Validation Dataset CQR – normalized (A) and not– normalized (B) were evaluated using the mean decreasing Gini coefficient. For each RF model, the confusion matrix, the accuracy, sensitivity, specificity and AUC – ROC values are reported.
